# Impact of a national guideline on use of knee arthroscopy: An interrupted time-series analysis

**DOI:** 10.1093/intqhc/mzz089

**Published:** 2019-11-14

**Authors:** Ali Kiadaliri, Dan Bergkvist, Leif E Dahlberg, Martin Englund

**Affiliations:** 1 Lund University, Faculty of Medicine, Department of Clinical Sciences Lund, Orthopaedics, Clinical Epidemiology Unit, Lund, Sweden; 2 Lund University, Faculty of Medicine, EPI@LUND (Epidemiology, Population studies, and Infrastructures at Lund University), Lund, Sweden; 3 Centre for Economic Demography, Lund University, Lund, Sweden; 4 Lund University, Faculty of Medicine, Department of Clinical Sciences Lund, Orthopaedics, Lund, Sweden; 5 Clinical Epidemiology Research and Training Unit, Boston University School of Medicine, Boston, MA, USA

**Keywords:** knee arthroscopy, degenerative knee disease, interrupted time series, Sweden

## Abstract

**Objective:**

To assess the impact of the Swedish health authority recommendation against the use of knee arthroscopy in patients aged ≥40 years with knee osteoarthritis (OA).

**Design:**

Interrupted time series analysis.

**Setting:**

Public health care in Skåne region.

**Participants:**

Patients aged ≥40 years who underwent knee arthroscopy from January 2010 to December 2015.

**Intervention(s):**

National guideline’s recommendation against the use of knee arthroscopy in patients with knee OA.

**Main Outcome Measure(s):**

1) proportion of patients aged ≥40 years with a main diagnosis of Knee OA and/or degenerative meniscal lesions (DML) who underwent knee arthroscopy, and 2) overall knee arthroscopy rate per 100,000 Skåne population aged ≥40 years.

**Results:**

A total of 6,155 knee arthroscopy were performed among people aged ≥40 years during study period. Of 42,044 patients with Knee OA/DML, 3,728 had knee arthroscopy. The recommendation was associated with reductions in the use of knee arthroscopy and two years after the recommendation, there was a reduction of 28.6% (95% CI: 9.3, 47.8) and 34.7% (23.9, 45.4) in proportion of Knee OA/DML patients with knee arthroscopy and the overall knee arthroscopy rate, respectively, relative to that expected if pre-recommendation trend continued. Our sensitivity analysis showed that the use of total knee replacement was stable over the study period.

**Conclusion:**

The national recommendation was associated with reduction in use of knee arthroscopy in public health care in southern Sweden. However, still 4.5% of these patients underwent knee arthroscopy in 2015 implying that more efforts are required to achieve the recommended target.

## Introduction

Knee arthroscopy is a commonly performed orthopaedic procedure in management of degenerative knee disease including degenerative meniscal lesions [1]. However, degenerative meniscal lesions are increasingly regarded as an early sign of knee osteoarthritis (OA) with no direct effect on pain [2] and there has been accumulating evidence suggesting no additional benefits of knee arthroscopy in these patients above placebo or non-surgical management over the last decade [3–9]. Currently international treatment guidelines generally recommend against knee arthroscopy only for patients with radiographic evidence of knee OA and they have variable recommendations regarding knee arthroscopy in patients with degenerative meniscal lesions without radiographic evidence of knee OA [10, 11]. In Sweden, the Board of Health and Welfare issued the first national guideline for musculoskeletal disorders in 2012 [12]. Aimed at decision makers and professionals, the guideline includes recommendations on osteoporosis, osteoarthritis and inflammatory rheumatic diseases. Based on available evidence at the time, the guideline concluded that knee arthroscopy in OA does not have a better effect on pain, function and quality of life than placebo treatment and hence recommended against the use of knee arthroscopy in patients with knee OA [12]. A potential unintended outcome of this recommendation is a redistribution of diagnostic codes from knee OA to other causes such as degenerative meniscal lesions [13]. In the current study, we aimed to assess the impact of this recommendation on the use of knee arthroscopy in public health care in southern Sweden.

## Method and Material

### Swedish healthcare system

Sweden has a publicly funded universal health care system, which is divided into three levels: the national (central government), regional (the 21 country councils), and local (the 290 municipalities) [14]. The central government, through the Ministry of Health and Social Affairs, is responsible for overall health care policy [14]. The county councils have the responsibility for the funding and provision of health care services. The municipalities are responsible for the provision of care for elderly, people with disabilities, and those in need of long-term mental health care [14, 15]. The National Board of Health and Welfare is one of the eight government agencies directly involved in the healthcare system. In addition to act as the licensing authority for health care staff, the National Board of Health and Welfare is responsible to provide, in collaboration with other actors, evidence-based guidelines for the care and treatment of patients with serious chronic diseases [14].

### Study Population and Setting

We performed a register-based study in the southernmost region of Sweden, Skåne, with a population of about 1.3 million in 2014 (13.2% of the Sweden’s population). We used the Swedish Population Register and the Skåne Health Care Register (SHR) to identify all residents of the region aged ≥40 years who at any time during 2010–2015 had a principal diagnosis of knee OA (the International Classification of Diseases, 10th revision [ICD-10] code M17) or derangement of meniscus due to old tear or injury (typically degenerative meniscal lesions, ICD-10 code M23.2) recorded by a physician (within primary or secondary care). We included degenerative meniscal lesions to avoid underestimation of arthroscopies due to OA because some patients who undergo arthroscopy have symptoms that are mainly due to OA even though degenerative meniscal lesions are reported as the main diagnosis. We also extracted data on performed procedures in the Skåne region from the SHR during 2010–2015 using the Swedish version of NOMESCO Classification of Surgical Procedures: arthroscopic or endoscopic exploration of knee (NGA11), arthroscopic or endoscopic total excision of meniscus in knee (NGD01), arthroscopic or endoscopic partial excision of meniscus in knee (NGD11), and arthroscopic or endoscopic partial excision of joint cartilage in knee (NGF31). These procedures were those that were recommended “not to use” in the national guideline and are considered knee arthroscopies in this study.

### Intervention and Outcomes

The publication of the national guideline for musculoskeletal diseases in May 2012 was considered as intervention in this study. Using data extracted from the SHR from 2010 to 2015, we defined two outcomes: 1) the proportion of patients aged ≥40 years diagnosed with a main diagnosis of knee OA/degenerative meniscal lesions who underwent knee arthroscopy, and 2) knee arthroscopy rate (irrespective of main diagnosis) per 100,000 Skåne population aged ≥40 years. This latter outcome would account for any potential redistribution of diagnosis codes. These outcomes were calculated bimonthly from 1 January 2010 to 31 December 2015.

### Statistical analysis

We used interrupted time-series analysis (ITSA) to estimate changes in the level and the pre-existing trend of study outcomes caused by the recommendation while controlling for pre-recommendation level and trend [16]. We estimated the following linear segmented regression model:

$$ {Y}_t={\beta}_0+{\beta}_1 Time+{\beta}_2 Intervention+{\beta}_3\ Intervantion\ast time+{\varepsilon}_t $$

where Y_t_ is the outcome at time point t, Time indicates the time since the start of study, Intervention is a dummy variable indicating pre-(coded 0) and post-recommendation (coded 1) period, and ε_t_ shows the error term. In this model, β_0_ estimates the baseline level of outcome at the beginning of the study, β_1_ represents the underlying pre-recommendation trend, and β_2_ and β_3_ estimate, respectively, the level and trend change following the recommendation [16]. To account for the lag between the publication of guideline and its implementation in practice, we considered a 6-month “phase-in” period and 3 bimonthly data points for this period were excluded from analysis. Therefore, we had 14 bimonthly data points before (January 2010–April 2012) and 19 bimonthly data points (November 2012–December 2015) after the recommendation. All models were assessed and controlled for seasonality using seasonal dummies. In addition to estimate changes in level and trend, we also estimated absolute and relative changes (with 95% confidence intervals) [17] in outcomes in two years after the recommendation (that is, in May 2014) compared with what would have been observed if pre-recommendation trend continued (counterfactual scenario).

The main threat to validity in an ITSA is *history*- the possibility that events co-occur with intervention could have caused the observed changes in outcome [18]. To account for this threat, we assessed changes in two outcomes that were not expected to be influenced by the recommendation: the proportion of patients diagnosed with knee OA/degenerative meniscal lesions aged ≥40 years who underwent a total knee replacement (TKR, NOMESCO codes of NGB29, NGB39, and NGB49), and TKR rate per 100,000 Skåne population aged ≥40 years. We also assessed the impact of increasing the “phase-in” period from six months to one year in a sensitivity analysis. We used the “itsa” command [19] in STATA (Version 15MP; Stata Corporation, College Station, TX, USA) which estimates the model using ordinary least-squares (OLS) with Newey–West standard errors to handle autocorrelation and heteroscedasticity.

**Table 1:** Summary statistics by intervention phases

	Pre-recommendation	Phase-in period	Post-recommendation
All patients (N = 42,044)			
n	18,308	6,144	27,767
Age, mean (SD)	65.7 (12.6)	66.1 (12.3)	66.5 (12.4)
Women, %	56.8	57.5	57.4
Diagnosed only with knee OA, %^a^	83.2	82.1	84.5
Diagnosed only with DML, %^b^	10.1	8.2	9.0
Diagnosed with knee OA and DML, %^c^	6.7	9.7	6.5
Proportion with knee arthroscopy, %	9.3	5.7	6.5
No. of knee arthroscopies	2682	562	2911
NGA11, no. (%)^d^	502 (18.7)	110 (19.6)	449 (15.4)
NGD01, no. (%)^d^	2 (0.1)	0 (0)	5 (0.2)
NGD11, no. (%)^d^	1583 (59.0)	328 (58.4)	1756 (60.3)
NGF31, no. (%)^d^	595 (22.2)	124 (22.0)	701 (24.1)
Mean (SD) no. of knee arthroscopies per month	191.6 (33.6)	187.3 (41.8)	153.2 (41.6)
Knee arthroscopy rate in Skåne population^e^	30.5	29.5	23.6

^a^ Patients aged ≥40 years with a main diagnosis of knee osteoarthritis (OA): ICD-10 code M17.

^b^ Patients aged ≥40 years with a main diagnosis of degenerative meniscal lesions (DMT): ICD-10 code M23.2

^c^ Patients aged ≥40 years with main diagnoses of both knee OA and degenerative meniscal tear (in separate healthcare contacts).

^d^ NGA11:arthroscopic or endoscopic exploration of knee, NGD01:arthroscopic or endoscopic total excision of meniscus in knee, NGD11:arthroscopic or endoscopic partial excision of meniscus in knee, and NGF31:arthroscopic or endoscopic partial excision of joint cartilage in knee.

^e^ Knee arthroscopy rate per 100,000 Skåne population aged ≥40 years regardless of diagnosis.

**Table 2:** Results of interrupted time series with segmented regression models

	Knee arthroscopies		Total knee replacement	
	Percent^a^	Operation rate^b^	Percent ^a^	Operation rate^b^
Pre-recommendation trend	−0.02 (−0.10, 0.05)	0.37 (0.07, 0.68)	−0.04 (−0.13, 0.05)	0.25 (0.08, 0.41)
Change in level	−0.55 (−1.46, 0.36)	−5.52 (−10.38, −0.66)	0.73 (−0.59, 2.05)	1.14 (−2.37, 4.66)
Change in trend	−0.12 (−0.20, −0.03)	−1.01 (−1.46, −0.57)	−0.04 (−0.16, 0.09)	−0.02 (−0.32, 0.29)
Intercept	5.67 (5.10, 6.24)	30.00 (28.08, 31.91)	9.89 (9.10, 10.69)	37.80 (36.61, 38.98)
Post-recommendation trend	−0.14 (−0.17, −0.10)	−0.64 (−0.97, −0.31)	−0.07 (−0.16, 0.01)	0.23 (−0.03, 0.49)
Absolute change in two years (May 2014)	−1.47 (−2.90, −0.04)	−13.64 (−20.11, −7.16)	+0.44 (−1.24, 2.12)	+1.01 (−2.77, 4.80)
Relative change in two years (May 2014)	−28.6% (−47.8, −9.3)	−34.7% (−45.4, −23.9)	+5.0% (−13.9, 23.9)	+2.3% (−6.1, 10.7)

All models adjusted for seasonality.

^a^ Percent of patients aged ≥40 years with a main diagnosis of knee osteoarthritis/degenerative meniscal tears who underwent knee arthroscopy.

^b^ Operation rate per 100,000 Skåne population aged ≥40 years.

## Results

We identified a total of 6,155 knee arthroscopies (regardless of main diagnosis, Table 1) performed among people aged ≥40 years during the study period, of which 59% were arthroscopic or endoscopic partial excision of meniscus in knee (NOMESCO Classification code NGD11). Of 42,044 patients with knee OA/degenerative meniscal lesion, a total of 3,728 (8.9%) patients had a knee arthroscopy over the study period. The proportion of patients with knee OA/degenerative meniscal lesion who underwent knee arthroscopy declined from 9.3% in pre-recommendation to 6.5% in post-recommendation period. From pre- to post-recommendation period, the overall knee arthroscopy rate decreased from 30.5 to 23.6 per 100,000 Skåne population aged ≥40 years.

**Figure 1:**
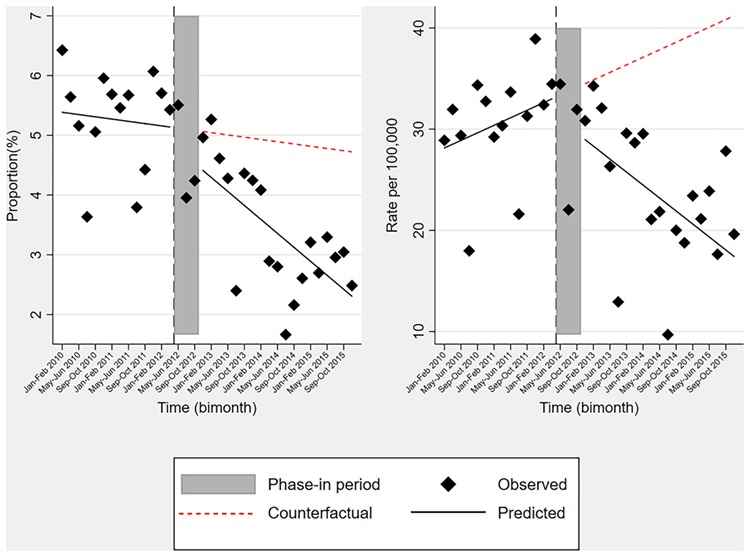
Interrupted time series analyses of the proportion of patients with knee osteoarthritis/degenerative meniscal tear who underwent knee arthroscopy (left), and knee arthroscopy rate per 100,000 Skåne population (right), 2010–2015.

The segmented regression analysis suggested that, after adjustment for seasonality, while there was no immediate change in proportion of knee OA/degenerative meniscal lesion patients with knee arthroscopy, it declined by 0.14% (95% CI: 0.10, 0.17) bimonthly after the recommendation (change in trend, Table 2, Figure 1). Two years after the recommendation, the proportion of knee OA/degenerative meniscal lesion patients with knee arthroscopy declined by 1.5% (0.0, 2.9), representing a relative reduction of 28.6% (9.3, 47.8) relative with expected if pre-recommendation trend continued.

**Figure 2:**
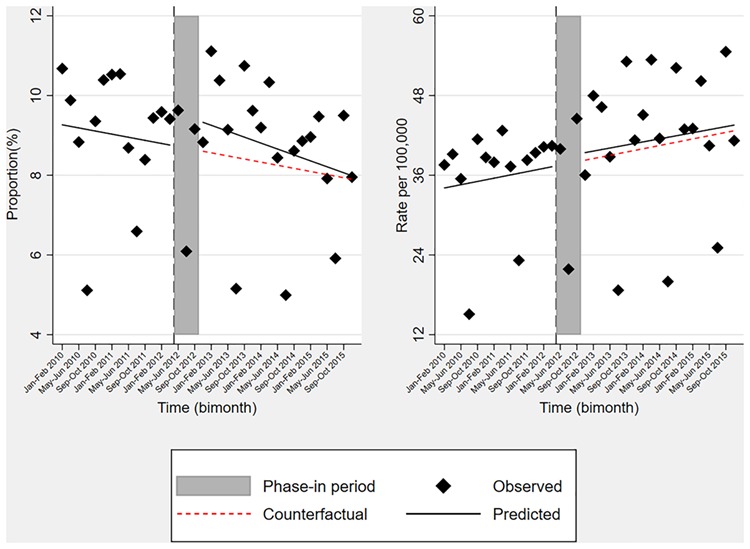
Interrupted time series analyses of the proportion of patients with knee osteoarthritis/degenerative meniscal tear who underwent total knee replacement (left), and total knee replacement rate per 100,000 Skåne population (right), 2010–2015.

Prior to the recommendation, the knee arthroscopy rate per 100,000 population aged ≥40 was increasing by 0.4 (0.1, 0.7) bimonthly, but *after* the recommendation it was decreasing by 0.6 (0.3, 0.9) bimonthly. In addition, we observed an immediate decline of 5.5 (0.7, 10.4) per 100,000 population aged ≥40 in knee arthroscopy rate after the recommendation. These changes translated in the absolute and relative reductions of 13.6 (7.2, 20.1) and 34.7% (23.9, 45.4), respectively, in two years after the recommendation. The recommendation did not have any impact on the use of TKR (Table 2, Figure 2). Furthermore, expanding the “phase-in” period from 6-month to 12-month did not essentially alter our findings (Table S1 in supplement).

## Discussion

Our results suggests that the publication of Swedish national guideline for musculoskeletal diseases was associated with reductions in the use of knee arthroscopy in public health care in southern Sweden. Two years following the guideline publication, there was a 35% reduction in knee arthroscopy rate in the general population relative to that expected if pre-recommendation trend continued.

Since publication of two randomized clinical trials reporting no additional benefit of knee arthroscopy compared with placebo or nonsurgical treatment in degenerative knee disease [7, 8], several studies reported that while knee arthroscopy declined among people with knee OA, it rose at the population level [13, 20–22]. This finding implies that the publication of clinical trials possibly led to a redistribution of diagnostic codes from knee OA to e.g. meniscal lesions [13, 22]. Actually, several studies documented large increases in diagnosis of meniscal lesions and arthroscopy for meniscal lesions over time [22–24]. On the other hand, in line with our study, several authors reported reductions in the use of knee arthroscopy following implementation of a policy or guideline [25–27]. This highlights the importance of knowledge translation, e.g. translating the scientific evidence into clinical guidelines and policies by organizations and national health authorities. The recommendation in the Swedish guideline was based on a review of the literature and concluded that in patients aged ≥40 with knee OA, knee arthroscopy provides no greater effect on pain, function, and quality of life compared with placebo treatment [12]. Moreover, the procedure involves an elevated risk of complications including joint infection and deep vein thrombosis [12]. Therefore, the guideline recommended “to not use” knee arthroscopy among OA patients aged ≥40 and estimated that this would save about 25 million Swedish Krona annually [12]. It should be noted that despite the observed reduction in the use of knee arthroscopy in our study, still 4.5% of OA patients aged ≥40 patients underwent knee arthroscopy in 2015 implying that more efforts are required to achieve the recommended target.

No changes in the use of TKR following the recommendation further supports that the observed declines in the use of knee arthroscopy are likely associated with the publication of guideline, and is simply not a result of a general systematic change in surgery rates. However, we cannot rule out that other supplementary factors might have contributed to the observed reduction. For instance, in Sweden a treatment model known as “Better management of patients with OsteoArthritis (BOA)” (https://boa.registercentrum.se/) was initiated in 2008 in four regions of Sweden including Skåne to provide all patients with OA adequate information and exercise based on treatment guidelines *before* performing any surgery. This intervention has become more popular over time and the number of primary care facilities offering the program has increased. Moreover, the publication of the trial by Sihvonen et al. [9] in 2013 which questioned effectiveness of knee arthroscopy for patients with symptoms of a degenerative meniscus lesion but *without* knee OA might have also (by itself) influenced the use of knee arthroscopy. Of course, the latter cannot explain the observed immediate decline in knee arthroscopy rate which occurred before the publication of the study.

Several limitations of the current study should be acknowledged. First, our study is subject to problems inherent to administrative register data including misdiagnosis and coding errors. Second, we conducted our study in the Skåne region and the results might not be generalizable to other regions or Sweden as a whole. Third, due to low number of performed knee arthroscopy, we were unable to assess the impact of the recommendation among age and sex subgroups. Finally, we should also acknowledge that we are presently unable to ascertain the number of knee arthroscopies performed in private practises in the region and its potential change over time. Still, these clinics are rather few in the Skåne region, and not likely to substantially altered the observed results.

## Conclusion

Our results suggest a reduction in the use of knee arthroscopy in public health care in southern Sweden following the publication of a national guideline that recommended against the use of this procedure among patients with knee OA. Conducting the similar analysis using the data on a national level is a subject for future research.

## Funding

This work was supported by the Crafoord Foundation, the Greta and Kocks Foundation, the Swedish Research Council, and the Österlunds Foundation.

Ethical Considerations.

The study was approved by the Lund University Ethics committee (Dnr 2014/276).

## Supplementary Material

Supplement_mzz089
