# Supplementary material for: Impact of a national guideline on use of knee arthroscopy: An interrupted time-series analysis
Source: Int J Qual Health Care. 2019 Nov 14;31(9):G113–8. doi: 10.1093/intqhc/mzz089 (PMC7076349; doi:10.1093/intqhc/mzz089)
Supplement: Supplement_mzz089 [file supplement_mzz089.docx]

Table S1. Results of interrupted time series with changing the phase-in period from six months to one year.

|  | Knee arthroscopies | | Total knee replacement | |
| --- | --- | --- | --- | --- |
|  | Percent ^a^ | Operation rate ^b^ | Percent ^a^ | Operation rate ^b^ |
| Pre-recommendation trend | -0.02 (-0.10, 0.05) | 0.37 (0.07, 0.68) | -0.04 (-0.13, 0.05) | 0.25 (0.07, 0.42) |
| Change in level | -0.82 (-1.85, 0.22) | -8.04 (-13.26, -2.81) | 0.04 (-1.52, 1.60) | 0.27 (-6.59, 7.13) |
| Change in trend | -0.09 (-0.17, -0.00) | -0.83 (-1.34, -0.32) | -0.09 (-0.24, 0.07) | -0.40 (-1.05, 0.26) |
| Intercept | 5.66 (5.09, 6.23) | 29.91 (28.00, 31.81) | 9.92 (9.14, 10.70) | 37.77 (36.54, 39.00) |
| Post-recommendation trend | -0.11 (-0.15, -0.06) | -0.45 (-0.87, -0.04) | -0.13 (-0.25, 0.00) | -0.15 (-0.78, 0.48) |
| Absolute change in two years (May 2014) | -1.60 (-3.05, -0.15) | -14.56 (-21.04, -8.08) | +0.30 (-1.42, 2.03) | +0.55 (-3.91, 5.01) |
| Relative change in two years (May 2014) | -31.1% (-50.1, -12.1) | -37.1% (-47.6, -26.5) | +3.4% (-15.7, 22.5) | +1.2% (-8.6, 11.0) |

All models adjusted for seasonality.

^a^ Percent of patients aged ≥40 years with a main diagnosis of knee osteoarthritis/ degenerative meniscal tears who underwent knee arthroscopy.

^b^ Operation rate per 100,000 Skåne population aged ≥40 years.
